# The glassy state of crambin and the THz time scale protein-solvent fluctuations possibly related to protein function

**DOI:** 10.1186/s13628-014-0008-0

**Published:** 2014-08-16

**Authors:** Kristina N Woods

**Affiliations:** 1 Physics Department, Carnegie Mellon University, Pittsburgh 15213, PA, USA

**Keywords:** THz spectroscopy, Protein dynamics, Picosecond time scale protein fluctuations, Protein glass transition

## Abstract

**Background:**

THz experiments have been used to characterize the picosecond time scale fluctuations taking place in the model, globular protein crambin.

**Results:**

Using both hydration and temperature as an experimental parameter, we have identified collective fluctuations (<= 200 cm^−1^) in the protein. Observation of the protein dynamics in the THz spectrum from both below and above the glass transition temperature (T_g_) has provided unique insight into the microscopic interactions and modes that permit the solvent to effectively couple to the protein thermal fluctuations.

**Conclusions:**

Our findings suggest that the solvent dynamics on the picosecond time scale not only contribute to protein flexibility but may also delineate the types of fluctuations that are able to form within the protein structure.

## Background

Proteins are essential components of all living organisms. It has long been recognized from both experimental and theoretical studies that substantial structural fluctuations occur in proteins. And consequently, these fluctuations are in some way essential for biological activity. Crambin is a highly hydrophobic and water insoluble protein, consisting of only 46 amino acids [1]. It occurs in the seeds of the plant *Crambe abyssinica* and belongs to the thionin family of membrane-active plant toxins. The role of the protein is still largely unknown, although it has been shown to exhibit extensive sequence homology with a family of membrane-active plant toxins [2]. For the most part, the residues in crambin have not been linked to a specific chemical activity, thus this may suggest that its natural function is determined for the most part by its structure, shape, or surface properties.

The crystal structure of crambin has been resolved [3],[4], but only recently at an exceptionally high resolution [3],[5] (0.48 A). In addition, extensive Neutron diffraction [5] and NMR studies [6],[7], in conjunction with theoretical investigations, have contributed significantly to the structural foundation of crambin. For this reason crambin is widely used as a model for protein computational studies as well as for the development of methodology used for assessing protein structure [8]. Likewise, the dynamical properties of crambin have also been a focal point of interest. One of the more intriguing aspects of crambin dynamics is the nature of the glass transition that occurs at about 200 K (−73 C). Many proteins and other amorphous solids have been found to undergo this transition, and its presence has been linked with the loss of large-scale collective motions of bonded and non-bonded groups of atoms in the system [4]. In proteins the change in dynamics that occurs during the transition has been particularly intriguing because the internal motions that are affected are often those that have been conjectured to be important for protein function [9]-–[13]. For instance, the large-scale collective motions in proteins are often viewed as transitions from one distinct protein conformational state to another and typically occur on fairly long time scales ranging from milliseconds to seconds. While the fast fluctuations within or between a specific protein conformation take place on a much shorter time scale (femtoseconds to picoseconds) and act as preliminary steps that guide the longer time scale conformational changes. These fast, thermal fluctuations in proteins would be visible in the THz region of the spectrum. Furthermore, there appears to be a growing number of studies [4],[14]-–[17] that have unequivocally demonstrated that the solvent is in some way intrinsically coupled with the fast dynamics of some proteins that are activated during the transition.

One question that we would like to begin to contemplate through the work presented in this manuscript is the type of distinctive information that can be obtained from THz time scale measurements on relevant protein motions that arise during the onset of the protein glass transition; and consequently, their contribution to overall protein function? At this point, we are certainly in no position to answer this question on a comprehensive level, but we can begin to address this query in a limited manner by considering the response of the model protein to both temperature and its immediate surroundings as it progresses through this dynamical transition. It is widely accepted that protein picosecond time scale fluctuations below 200 K are mostly harmonic in nature [15],[18],[19], and under this premise, only able to explore a limited portion of the protein conformational landscape that maps out protein function [15],[20]. However, once the (glass) transition temperature has been reached, the protein atoms begin to vibrate anharmonically and they are no longer confined to a restricted topography. Incidentally, the anharmonic fluctuations that develop within the protein are assumed to be greatly enhanced through interaction with the protein solvent [13],[21]-–[24]. To comprehend both the nature and origin of the detected modes in the THz spectrum of crambin, we have carried out experiments that chart the evolution of the protein fluctuations as the glass transition is crossed as both a function of temperature and hydration. In this case, the amount of solvent in the hydration shell is used as an experimental variable to assess how the picosecond time scale fluctuations arising in the hydration layer may couple to the relaxational degrees of freedom of the protein during the onset of the transition. And although the internal fluctuations of the protein are explored experimentally, in this investigation we also include a computational component to our analysis (by way of molecular dynamic (MD) simulation) to aid in the experimental interpretation of the detected modes. Our aim is to provide a clearer picture about the molecular mechanism of the actual transition in a model protein system and perhaps a backstory about the possible role that these motions play in protein function.

## Results and discussion

### The glass transition in crambin and protein intrinsic modes

From the MD simulations performed in our analysis of crambin, we have determined that the surface residues in the turn regions of the protein have the largest amplitude dynamics in the THz region of the simulation spectrum. And additionally, the actual number of solvent molecules in the hydration layer also appears to have a strong influence on both the amplitude and the vibrational frequency of the protein fluctuations that arise due to contact with the surrounding water molecules. For this reason, in this investigation we have prepared two distinct samples of crambin with differing number of water molecules within the hydration shell. Our objective for the preparation of the individual samples is to observe how solvent coupling may affect the instrinic dynamics of a globular protein on the picosecond time scale. In one sample, there are only an adequate number of water molecules to fill the first or primary layer of the hydration shell. The other sample possesses a greater number of water molecules such that there are a sufficient number to completely fill both the *first* and the *second* hydration layer within the shell. From this point on, the samples will now appropriately be referred to as the low hydration sample (LHS) and the high hydration sample (HHS) for the remainder of the manuscript.

Moreover, we have prepared equivalent samples for the MD simulations carried out in this investigation. In the simulations, the hydration layer of the low hydration crambin sample contains water molecules to within a 3.8 Å distance from the protein surface, which is also consistent with the minimum number of water molecules that have been found to be necessary for hydrating both polar and apolar amino acid surface groups in the primary hydration layer of proteins [25]. It also corresponds with the estimated number of water molecules in the hydration shell of our experimentally prepared low hydration crambin sample. The higher hydration sample from the MD simulation contains water molecules that extend out to 8.0 Å distance from the protein surface. The higher hydration MD simulation sample of crambin also approximates the measured number of water molecules in the analogous experimental sample and has been shown to relate to a biologically relevant hydration level [26] in other globular proteins. Although, it is important to note that recent experimental evidences suggests that the hydration layer around a protein has dynamics distinct from the bulk water that extends as far as 10 Å from the protein surface [27] with the effects on the surrounding water network reaching beyond a distance of 20 Å [28] from the protein periphery.

**Figure 1:**
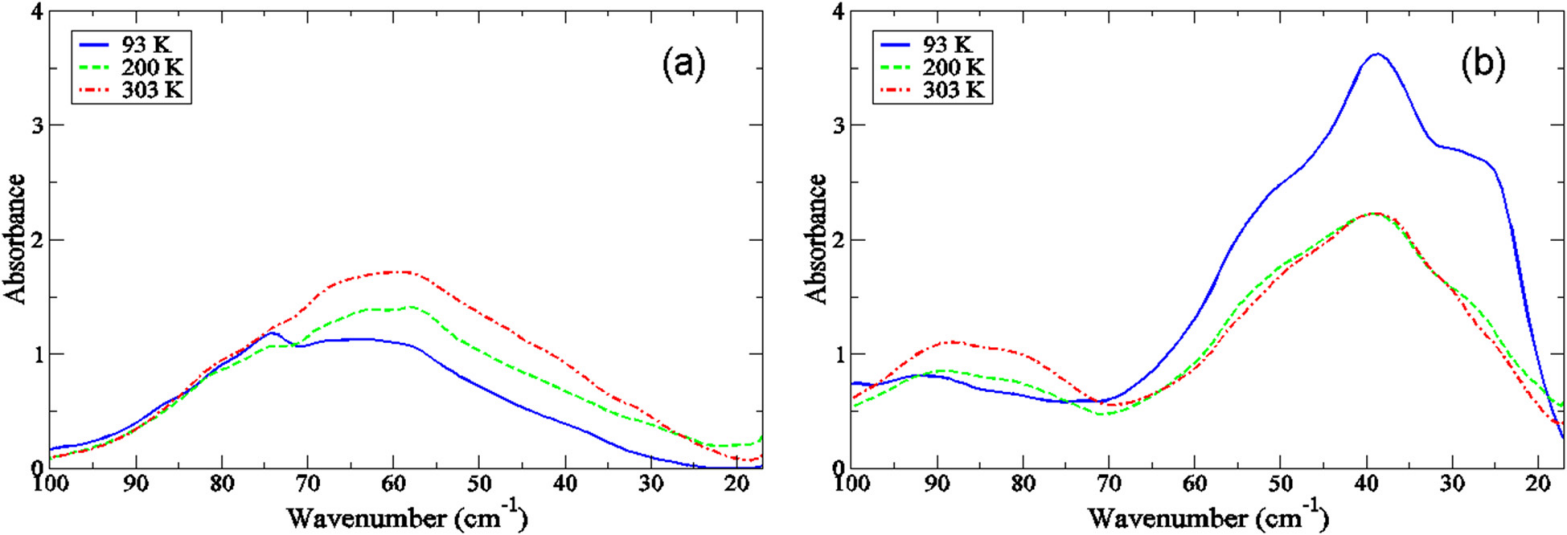
**Experimental THz spectrum of (a) the HHS and (b) the LHS of crambin at 93 K (blue solid line), 200 K (green dotted line), and 303 K (red dotted-dashed-dotted line) in the 20 – 100 cm**^**−1**^**spectral region.**

Results from numerous studies have indicated that the fast solvent dynamics are capable of manipulating protein function in the sub-nanosecond regime [4],[15],[27],[29],[30]. Although, the extent to which water structure on a fast time scale may directly effect the fluctuations of a protein are still largely unknown. In this work, we plot the experimental THz spectrum of crambin as a function of both hydration and temperature in the 20–100 cm^−1^ spectral region in Figure 1. We conjecture that development of the peaks in the low-frequency region of the two samples may be key for ascribing a hypothesis about how the modes activated during the glass transition may relate to function or at least (bio)activity. For one thing, we notice that the shape and individual peaks in the two samples are appreciably dissimilar. At first glance, the HHS spectrum in Figure 1a appears to contain only one large, major peak centered at about 60 cm^−1^. Based on the peak’s temperature dependence and previous investigations on globular proteins [31],[32], we initially speculate that ~60 cm^−1^ centered mode stems from localized, internal side-chain fluctuations that are indirectly connected with the hydration water dynamics on the picosecond time scale. In the LHS spectrum (Figure 1b), we identify a similar type of side chain fluctuation at about 90 cm^−1^. Below the glass transition temperature, the LHS spectrum also exhibits prominent peaks at approximately 50, 40, and 25 cm^−1^ in the low-frequency spectral region. The 50 cm^−1^ and 40 cm^−1^ modes are likely *delocalized* backbone torsions that are directly intertwined with the fast water dynamics taking place in the protein hydration shell [31],[33], while the ~25 cm^−1^ mode in the LHS spectrum is close in frequency to the reported boson peak from other investigations [34]-–[38] on protein glassy dynamics. Additionally, a solvent-induced dispersive mode has been identified in THz time scale scattering measurements in globular proteins [14],[39] at a similar frequency (~25 cm^−1^). Hence, we tentatively attribute the peak at 25 cm^−1^ identified in this investigation to a global backbone torsion that is coupled with α-helical side chain fluctuations. And like the 40 cm^−1^ and 50 cm^−1^ collective vibrational modes in the LHS spectrum, the 25 cm^−1^ peak appears to be connected with the fast hydration dynamics in the solvent.

### Hydrogen-bonding (H-bonding) fluctuations investigated from MD simulation

#### Probing protein-protein H-bonding fluctuations in the low frequency spectral region from MD simulation

**Figure 2:**
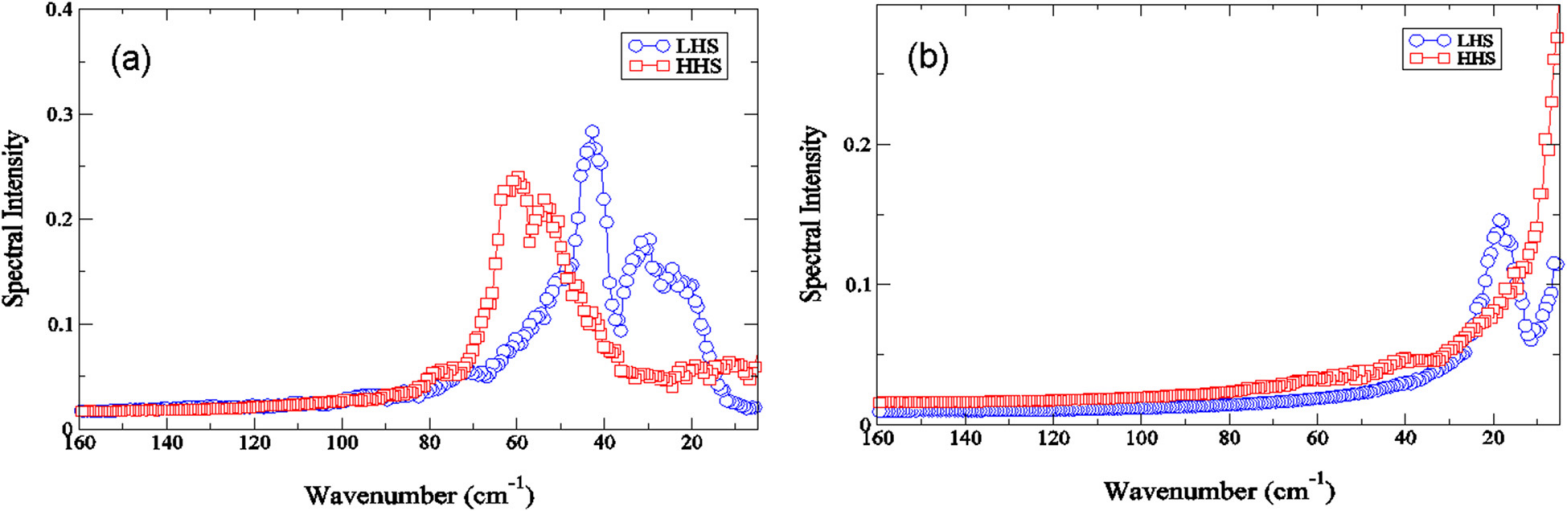
**Inter- and intra-protein hydrogen bond (H-bond) correlations. (a)** Spectrum of intra-protein (protein-protein) H-bond correlations of the HHS (red open squares) and the LHS (blue open circles) of crambin from the MD simulations at 300 K. **(b)** Spectrum of solvent-protein H-bond correlations of the HHS (red open squares) and the LHS (blue open circles) of crambin from the MD simulations at 300 K.

Using the MD simulations of crambin in the two differing hydration levels, we find that the internal fluctuations in the two systems appear to strongly reflect the dynamics of the global motions that we detect experimentally in Figure 1. It has been shown previously, that fluctuations in both the protein intramolecular and intermolecular [40] hydrogen bond (H-bond) network have an important role in maintaining the tertiary structure of proteins as well as the activity [25],[41]. For instance, from our MD simulations on crambin, we find that the protein *intramolecular* H-bond fluctuations in the LHS sample in Figure 2a produce discernible peaks in the ≤ 100 cm^−1^ region of the spectrum at approximately 25 cm^−1^ and 45 cm^−1^. In the HHS crambin system there is a single prominent peak in the MD simulation spectrum that is centered at about 65 cm^−1^. In both samples, the internal fluctuations appear to be highly dominated by protein intramolecular H-bonding.

#### Probing solvent-protein H-bonding fluctuations in the low frequency spectral region from MD simulation

Interestingly, analyzing the role of solvent coupling to the low-frequency protein dynamics from the MD simulation (Figure 2b), we also find differences when contrasting the two different hydrated crambin samples. In Figure 2b, it becomes apparent that the LHS crambin interaction with the solvent molecules in the hydration shell is more pronounced when compared with that of the HHS. Specifically, protein-solvent interactions create a prominent translational peak at 25 cm^−1^ in the LHS while comparable solvent-induced fluctuations in HHS are generally revealed to be much weaker.

### Deuterium exchange and the effect of solvent dynamics on the experimentally detected THz time scale protein global modes

**Figure 3:**
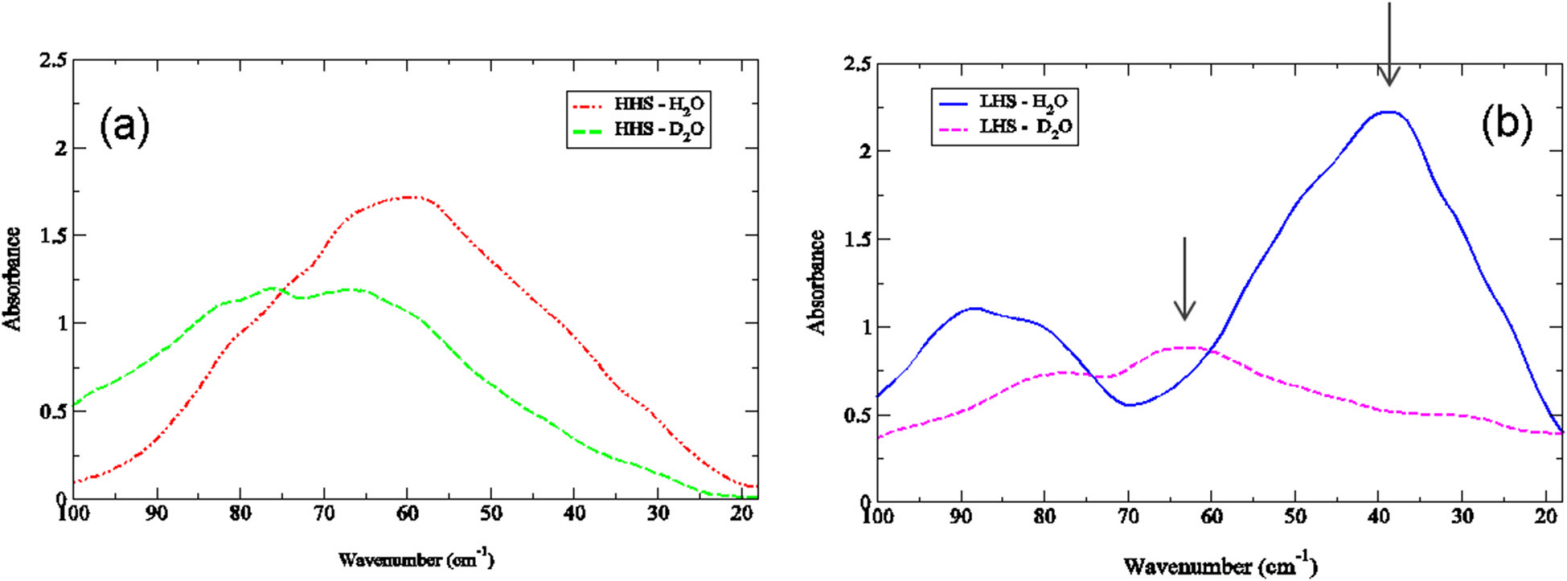
**Experimental THz spectra of (a) the HHS of crambin in H**_**2**_**O (red dot-dashed-dotted line) and D**_**2**_**O (green long dashed line) at 300 K and (b) the LHS of crambin in H**_**2**_**O (blue sold line) and D**_**2**_**O (magenta short-dashed line) at 300 K in the 20–100 cm**^**−1**^**spectral region.**

Experimentally, deuterium exchange can be used as a parameter for assessing the global protein modes that are most intertwined with the solvent dynamics on the picosecond time scale. In Figure 3a, we find only a weak correlation with the lowest frequency experimental mode in the HHS of crambin with the dynamics of water molecules in the protein hydration layer. We observe only a moderate shift in the most prominent mode in the HHS spectrum describing the protein global modes, while on the other hand, there is a definite, noticeable blue-shift of the peak initially positioned at about 40 cm^−1^ in H_2_O to approximately 65 cm^−1^ in D_2_O in the LHS in Figure 3b. The large shift in peak position in the LHS that is accompanied by a large decrease in intensity suggests that the interaction with the solvent reflects an overall increase in rigidity of the protein structure in heavy water [33],[42]. We also find a small red-shift of the ~90 cm^−1^ mode in the LHS when the solvent is replaced with D_2_O and a negligible blue-shift of the lowest frequency mode from approximately 27 cm^−1^ to 30 cm^−1^ in D_2_O.

### Velocity autocorrelation function from MD simulation and its relationship to experimentally detected low-frequency global modes in crambin

**Figure 4:**
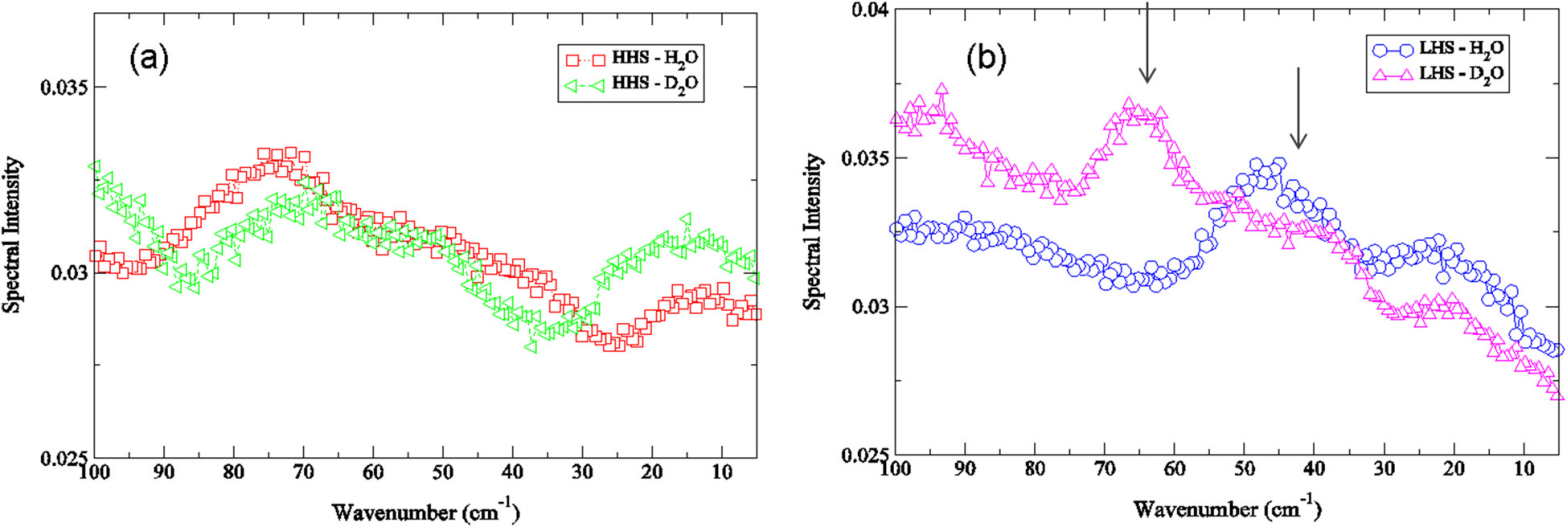
**Fourier Transform of the velocity autocorrelation function of the backbone atoms (a) of the HHS of crambin in H**_**2**_**O (red open squares) and D**_**2**_**O (green left-facing open triangles) from the MD simulation at 300 K and (b) the LHS of crambin in H**_**2**_**O (blue open circles) and D**_**2**_**O (magenta open triangles) from the MD simulation at 300 K.**

Velocity autocorrelation functions (VACFs) from MD simulations can be used to project out the underlying frequencies of the molecular processes associated with specific correlated motions detected in the simulation. Although we cannot directly compare the amplitude of these frequencies from simulation with the IR intensities detected from experiment, they have proven to serve as a useful guide for assessing the origin of the most prominent motions detected in the THz spectra of various proteins [32],[43],[44]. Along these same lines**,** we have elected not to compute the dipole autocorrelation function of the protein motion from the MD simulations, which could in theory be directly compared with experiment, due to the fact that the classical force-fields used in the simulations are not at a level of accuracy that would provide an accurate representation of the electronic properties of the protein. For instance, a plot of the Fourier Transform of the velocity autocorrelation function of the backbone motion of both crambin samples in H_2_O when contrasted with that in D_2_O from the MD simulation in Figure 4 are in line with our experimental observations in Figure 3. In Figure 4a, we find that deuterium exchange has a minor effect on the global backbone dynamics of the HHS crambin sample but a dramatic effect on the backbone fluctuations of the LHS (Figure 4b). Similar to what has been observed experimentally (Figure 3a-b), deuteration causes a clear blue-shift of the collective backbone motion of the protein although in this case, the intensity of the backbone mode from the MD simulation is relatively unaltered with the shift.

### Low frequency collective excitations in the interfacial hydration water of crambin identified from MD simulation

**Figure 5:**
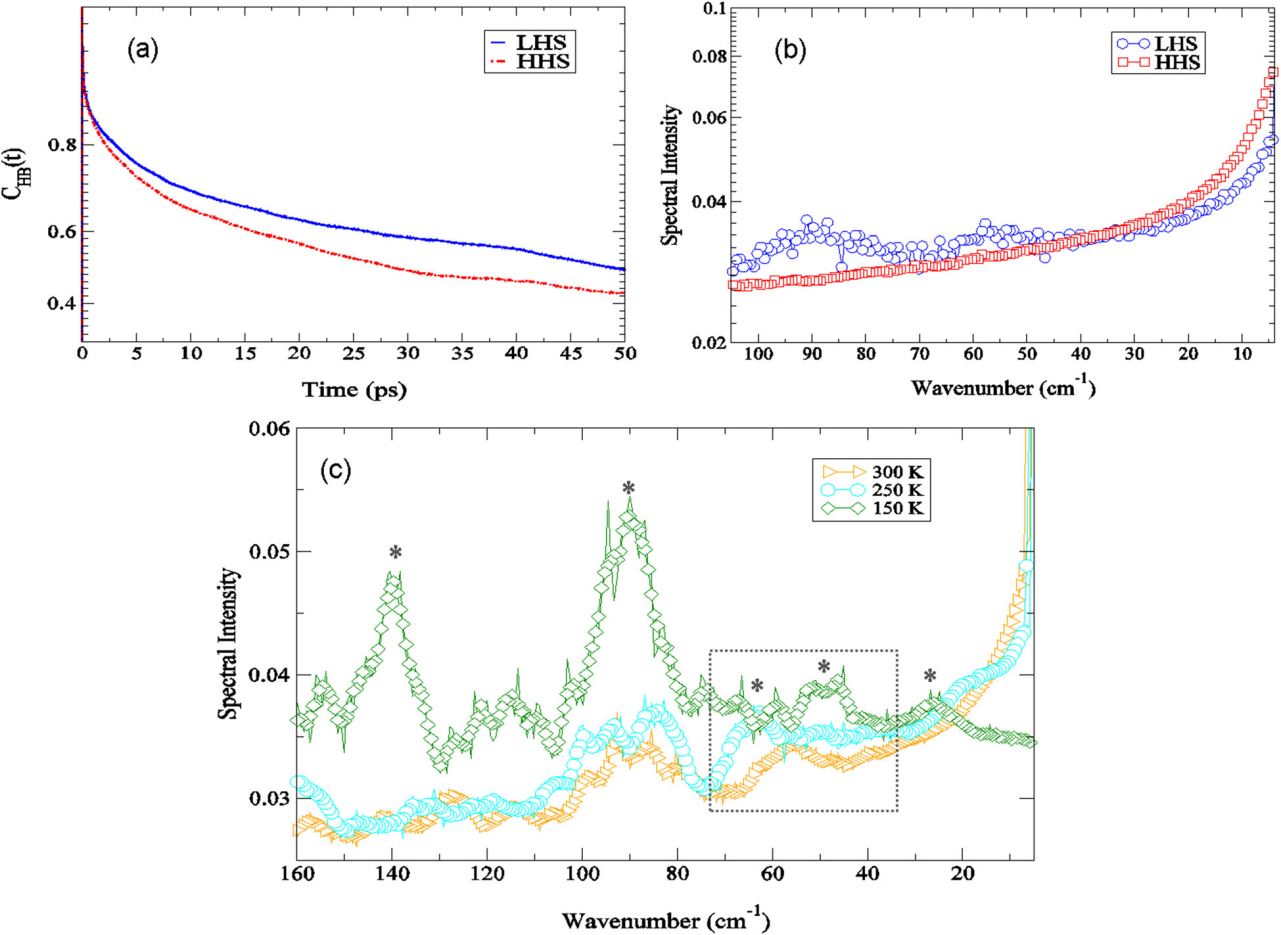
**Interfacial water-water hydrogen bond autocorrelaton function (HBACF). (a)** Interfacial water-water HBACF of water molecules at a distance of 3.8 Å from the protein surface in the LHS (blue solid line) and the HHS (red dash-dot-dotted line) of crambin at 300 K from the MD simulations. And **(b)** the corresponding Fourier Transform of the interfacial water-water HBACF [*from (a)*] of the LHS (blue open circles) and the HHS (red open squares) of crambin. **(c)** Spectrum of the interfacial water-water HBACF of the LHS of crambin at 150 K (green open diamonds), 250 K (cyan open circles), and 300 K (yellow open right-facing triangles) from the MD simulations.

Water hydrogen-bond time autocorrelation functions (HBACFs) have the potential to reveal information about local intermolecular structure and H-bonding lifetimes of water molecules in the protein hydration shell. Contrasting the LHS with the HHS interfacial water-water HBACF from the MD simulation in Figure 5a we find a faster decay rate of H-bonds among water molecules that reside in the HHS hydration shell when compared with water molecules contained within the LHS shell. The hydrogen bond lifetimes for interfacial water molecules in the LHS and the HHS hydration shell were found to have values of *τ* = 2.9 ps and *τ* = 2.6 ps, respectively. Perhaps the underlying differences in the lifetimes can be attributed to both the local structure of the water molecules in the shell as well as the contributing relaxation processes taking place within the water layer. In Figure 5b, we find that the motions reflected in the spectrum of the HBACF result in somewhat diffuse peaks in the LHS spectrum at about 55 cm^−1^ and 90 cm^−1^ in the ≤ 100 cm^−1^ spectral region, whereas the correlation spectrum of the HHS of crambin discloses no obvious peaks in the same region. With the hope of ascertaining more information about the actual nature of the motions detected in spectrum of the LHS HBACF, we have performed an analogous MD analysis as a function of temperature and analyzed the spectral features from the HBACF in an expanded spectral region. At the lowest temperature investigated (150 K) in the MD simulations, the spectrum of the H-bond time autocorrelation function of the LHS hydration water in the 10 – 160 cm^−1^ spectral region uncovers two prominent peaks at about 140 cm^−1^ and 80 cm^−1^ in Figure 5c. Both peaks have maximum intensity at low temperature and are either absent or greatly reduced at room temperature, suggesting that they arise from stretching motions that are most likely due to incorporation of water molecules into protein amino acid side-chain and backbone dynamics. At a lower frequency there is a barely perceivable shoulder in the spectrum at about 25 cm^−1^ that is red-shifted as the temperature increases and eventually disappears at the highest temperature investigated. Between 40 cm^−1^ and 70 cm^−1^ there are two bands that form in the HBACF spectra that have contrasting temperature dependencies. At 150 K there is a detectable peak at 50 cm^−1^ that slightly shifts to a higher frequency and decreases in amplitude as the temperature is increased. At 250 K another peak in the 40 – 70 cm^−1^ spectral region emerges at about 65 cm^−1^ that also decreases in intensity as the temperature is increased but moves to a slightly lower frequency. At 300 K both the 50 cm^−1^ and the 65 cm^−1^ H-bond time autocorrelation modes in the LHS solvent shell spectrum merge into a single peak that is centered at 55 cm^−1^. We are extremely interested in this ~50 cm^−1^ mode in the hydration HBACF spectrum because it could offer insight into the mechanism in which solvent modes couple to the low-frequency protein motions in crambin. We observed in the previous section that a peak close in frequency may be linked with coupling low-frequency motions of the protein to collective excitations involving water molecules in the hydration shell (Figures 3 and 4). Recent MD simulation investigations [45] on supercooled water using classical potentials have uncovered peaks at a similar frequency (*v* < 60 *cm*^−1^) that have been attributed to transverse acoustic-like phonon modes in the water network that are spatially extended over large length scales. Perhaps it is through these collective excitations that form in the solvent network at low hydration that the global protein motions in crambin are enhanced. Unfortunately, we are unable to disentangle the nature of the protein-solvent coupling mechanism simply from analyzing the experimental modes detected in the ≤ 150 cm^−1^ region of the spectrum. However, we are able to investigate an experimental region that is highly dominated by *liquid water* translation motion. The higher frequency excitations in water can be used to characterize the local (intermolecular) structure of water molecules in the hydration shell and may provide more information about how these intermolecular vibrational modes are tied with (i) the relaxation processes taking place in the hydration layer and (ii) the manner in which these intermolecular modes are able to couple with the instrinic dynamics of the protein.

### Experimental detection of THz translational modes in crambin hydration water

**Figure 6:**
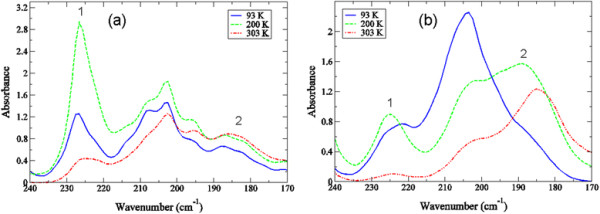
**Experimental THz spectrum of the translation region of water in the 170–240 cm**^**−1**^**spectral region of (a) the HHS and (b) the LHS of crambin at 93 K (blue solid line), 200 K (green dashed line), and 303 K (red dot-dash-dotted line).** The labels of (1) and (2) in the spectra of **(a)** and **(b)** refer to distinct intermolecular structures [46] of water that we have assigned at specific frequencies in the experimental spectra.

In Figure 6, there are three prominent peaks in the experimental THz spectrum in the 170 – 240 cm^−1^ spectral region that we attribute to water intermolecular vibrational modes. Peaks at approximately 225 cm^−1^ and 180 cm^−1^ have been identified in time-resolved OKE spectroscopy experiments [46] on water and have been ascribed to distinct intermolecular local configurations in the liquid water network. The local structure at 225 cm^−1^ corresponds to a highly tetrahedral water molecular arrangement while the lower frequency (~180 cm^−1^) structure relates to a compressed configuration with a distorted H-bond network. It has been proposed that relaxation induced reorganization between the two structures may potentially affect vibrational mode delocalization in liquid water that consequently, may also play an integral role in many different chemical and biological processes [45],[47],[48].

In Figure 6a, the HHS in the water translational region that we detect experimentally has a narrow, prominent band centered at about 225 cm^−1^ that initially grows in intensity as the temperature is increased but broadens and loses intensity at ambient temperature. Interestingly, the other two prominent peaks centered at approximately 205 cm^−1^ and 185 cm^−1^ in the HHS experimental spectra are somewhat temperature independent and do not change appreciably as the temperature is varied. Incidentally, on closer inspection, the band located close to 205 cm^−1^ in the HHS spectra appears to be comprised of a cluster of peaks rather than a single individual peak. The cluster includes peaks at approximately 195 cm^−1^, 202 cm^−1^, and 207 cm^−1^. The varied temperature dependency of the individual peaks in the cluster suggests that the bands may be composed of a mixture of water structures with differing levels of H-bond connectivity. It is interesting to note that cluster vibrations involving combinations of O-H●●● O stretching vibrations have been detected in both theoretical [49] and experimental [50] investigations in liquid water at around 200 cm^−1^.

The experimentally detected water translational bands in the LHS in Figure 6b feature peaks at a similar frequency when contrasted with the HHS but the temperature progression differs significantly. The intensity of the 225 cm^−1^ band is greatly reduced in the LHS. There is a minor change in peak intensity as the temperature is increased but at room temperature the band has almost completely disappeared from the spectrum. Intriguingly, both the 205 cm^−1^ and 185 cm^−1^ modes have very notable temperature dependencies in the LHS spectra. The 205 cm^−1^ dominates the spectrum at 93 K while there is only a shoulder at 185 cm^−1^. As the temperature is increased the peak at 205 cm^−1^ decreases in intensity whereas the 185 cm^−1^ grows in intensity and shifts to a slightly lower frequency. The singly resolved band at 205 cm^−1^ (rather than a cluster of vibrational modes) and its clear temperature dependency may indicate that the hydration shell in the LHS is comprised of a more uniform population of O-H●●●O stretching vibrations in general. However, since the nature of the mode(s) close to 205 cm^−1^ is presently uncertain in both crambin samples we will focus our efforts on trying to elucidate the characteristics of the 225 cm^−1^ and 185 cm^−1^ modes in the experimental translation region. Under this premise, we will assume that both the 225 cm^−1^ and 185 cm^−1^ modes are directly related to *distinct* water structures in the protein solvent shell. In this context, the experimental spectra suggest that in the HHS solvent there are a far greater number of water molecules arranged in a tetrahedral configuration at low temperature but under ambient conditions the number of water molecules in both “structured” and “distorted” H-bond configurations is almost equal. On the other hand, the LHS hydration shell is almost entirely comprised of unstructured water molecules above 200 K and further, the motion associated with these molecules in the shell appears to be anharmonic.

Interestingly, in both samples in Figure 6, the peak at ~ 225 cm^−1^ has a maximum amplitude at 200 K but at all temperatures investigated there is no shift in peak frequency suggesting that the fluctuation remains vibrational for all temperatures explored. We see a similar trend with the peak intensity at 185 cm^−1^ at 200 K in the LHS sample in Figure 6b, only in this case there is a clear red-shift of the peak frequency as the temperature is increased above 200 K, implying that the motion associated with the fluctuation is anharmonic [51]. Previous work on myoglobin using both experimental and computational simulations [9],[52],[53] have detected a dynamical transition above 180 K in the protein that results from a coupling of fast (picosecond time scale) local motions in the protein with slower, collective protein fluctuations. This coupling of motions is strongly interconnected with a glass-like transition that takes place in the protein hydration shell. Further, the picosecond time scale structural fluctuations in the protein that are activated during the transition are anharmonic in nature. Using these previous investigations on protein-solvent dynamics as a basis for a description of what we observe in our experimental studies on crambin, it is conceivable that the motion associated with the less structured water molecules in the LHS solvent spectrum in Figure 6b is directly tied with the long-range communication that we have detected experimentally in the protein under low hydration conditions (Figures 1 and 3). Under this premise, we speculate that the anharmonic dynamics of the solvent molecules within the hydration shell couple with protein motions and promote long-range coherence in the protein three-dimensional structure. In the HHS there seems to be an entirely different mechanism fostering water molecule intermolecular associations in the hydration shell. Based on the characteristics of the detected translation modes in Figure 6a, the motion of water molecules in the HHS hydration shell is dominated by more self-associating, localized dynamics that is not readily integrated into the relaxational processes of the protein. Feasibly this then begins to provide an explanation for the general absence of *collective* fluctuations in the HHS low frequency vibrational region that we have detected experimentally. If we assume that the possible water-mediated interaction sites on the hydrophobic protein surface are by nature somewhat limited, then an excess amount of solvent in the hydration shell may drive the water molecules present in the layer to maximize their interactions with each other rather than with the protein. Recalling that the interaction of the solvent has been conjectured to be central to the eventual development of protein anharmonic fluctuations above the glass transition, we note that in our experiments we have observed that the types of surface interactions that HHS shares with the solvent include mostly localized side-chain torsions that do not change appreciably during the transition (Figure 1). Experimentally, we detect only a small rise in protein global mode peak intensities as the temperature is increased above the glass transition region indicating that the detected vibrational modes in the HHS spectra are restrained to harmonic oscillations.

### Localized protein interactions and long-range communication in crambin

#### The origin of long-range communication in crambin?

**Figure 7:**
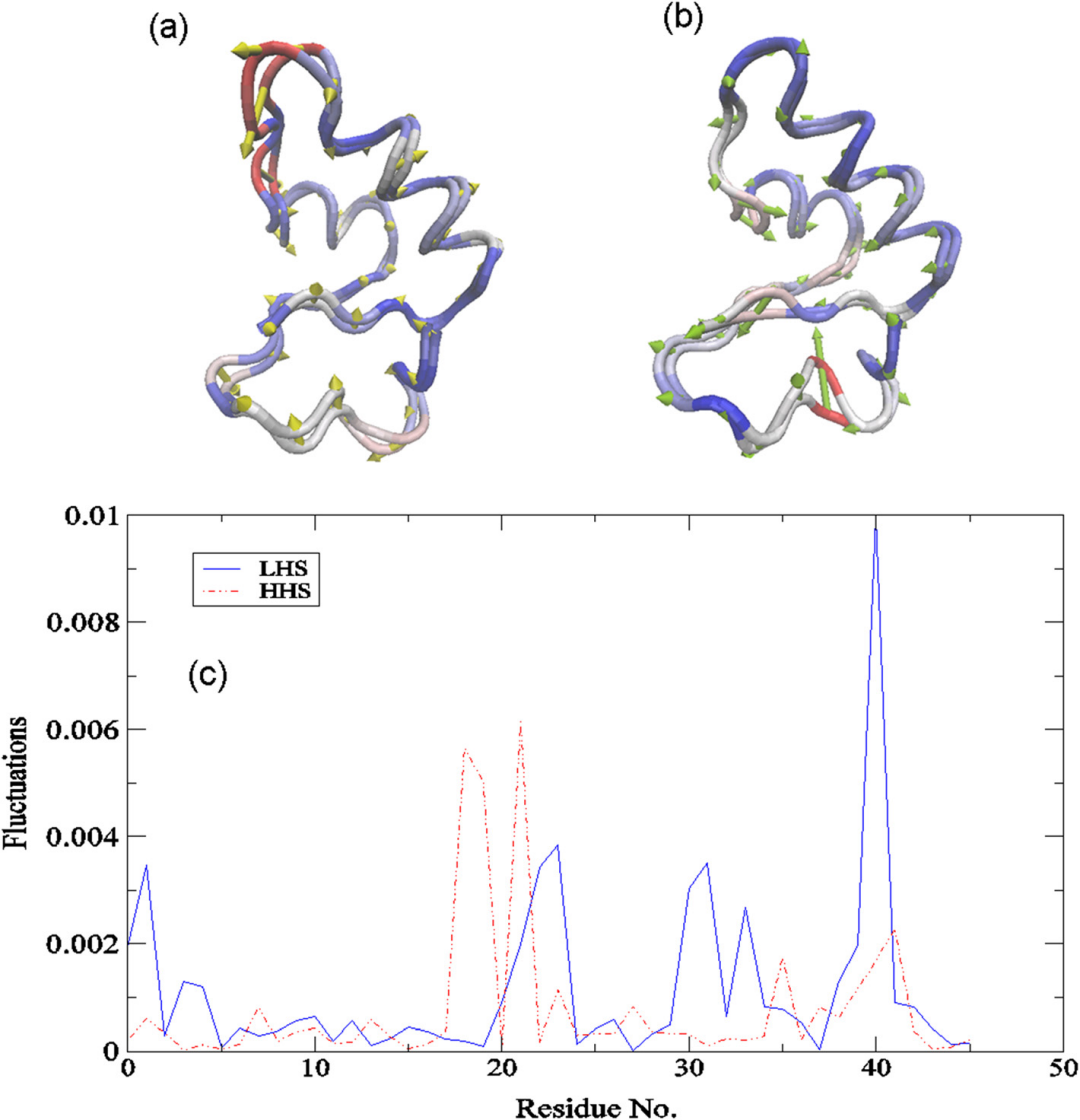
**Largest amplitude PCA mode.** Trace representation depicting the C_α_- motion of the largest amplitude PCA mode (PC1) of **(a)** the HHS and **(b)** the LHS of crambin from the MD simulation. Regions highlighted in red represent more flexible regions of the protein and regions highlighted in blue represent regions with less flexibility. And **(c)** the corresponding square fluctuations of the PC1 of the LHS (blue solid line) and the HHS (red dot-dash-dotted line) of crambin.

To explore the nature of the long-range communication of the protein as a function of hydration, we have conducted principal component analysis (PCA) on the MD simulations of the hydrated crambin systems. At first glance (Figure 7) it is clear that there are distinct differences in the collective fluctuations of the two hydrated protein samples. The depiction of the largest amplitude PCA mode (PC1) in Figure 7a – b clearly illustrates that the dominant motion in the HHS of crambin is decidedly localized whereas the LHS depicts a more collective motion. In Figure 7a we find that the solvent exposed loop (residues 19 – 23) is highly mobile in the HHS of crambin and this is reflected in the square fluctuation amplitude of the PCA mode in Figure 7c. In Figure 7b, PC1 of the LHS describes a network of correlated fluctuations that permeate through the core of the molecule, which is also evident in the plot of the square fluctuations along the PCA.

#### Experimental detection of “more” localized intra-protein interactions and the formation of protein long-range coherence pathways in crambin

**Figure 8:**
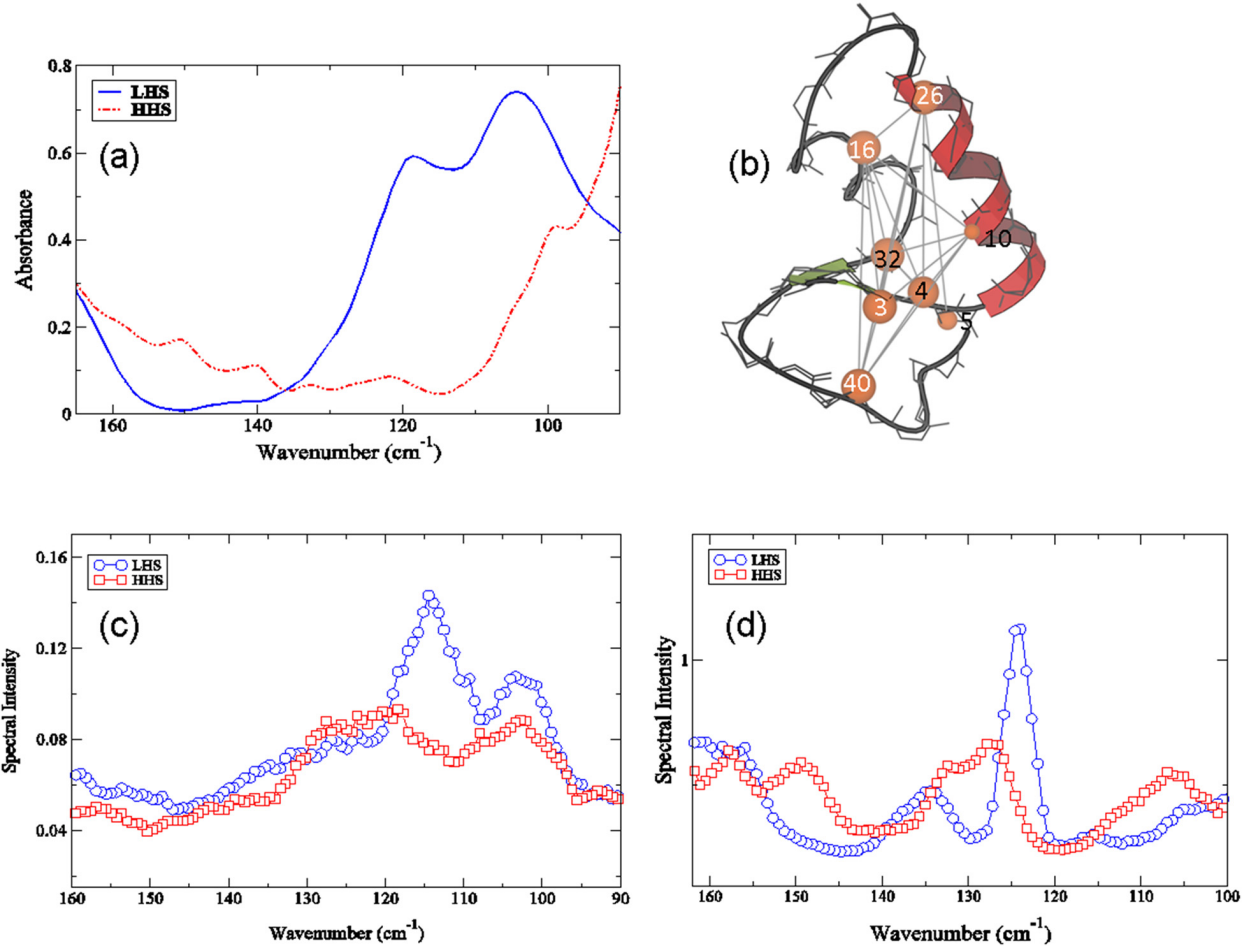
**Experimental THz spectra and long-range correlated fluctuations. (a)** Experimental THz spectrum of the LHS (blue solid line) and the HHS (red dotted-dashed-dotted line) of crambin in the 100 – 165 cm^−1^ spectral region at 303 K. **(b)** The network representation of the LHS of crambin depicting long-range correlated fluctuations in the protein detected in the MD simulations (using Equation 3). The nodes represent amino acids and the sizes of the nodes reflect the amplitude of the fluctuation. The edges between the nodes reflect correlated fluctuations and the thickness of the edges depict the extent of the correlation. The nodes labeled in white represent amino acids involved in disulfide bridges. **(c)** The Fourier Transform of the rotational autocorrelation function of the vector between the C_α_ atoms of residues Cys 16 and Cys 40 and **(d)** the spectrum of the velocity autocorrelation function of the correlated fluctuation involving Cys 16 and Cys 40 from the MD simulations.

Experimentally, we are able to detect *more* localized interactions taking place in the protein (such as a network of interactions that involve a subset of the molecule rather than the entire protein) in the 100 – 170 cm^−1^ spectral region. In Figure 8a a plot of both hydrated crambin samples in the 100 – 170 cm^−1^ experimental spectral region reveals two prominent modes in the LHS spectrum at about 105 cm^−1^ and 120 cm^−1^; while interestingly, the HHS spectrum lacks any large amplitude peaks in the same spectral region. In the HHS experimental spectrum we do observe several small-amplitude oscillations at approximately 105 cm^−1^, 120 cm^−1^, 135 cm^−1^, 140 cm^−1^, 150 cm^−1^, and 158 cm^−1^. The smaller amplitude modes in HHS spectrum most likely represent small-scale internal fluctuations that have been observed in previous THz studies [54]-–[56] on globular proteins and other peptides.

**Figure 9:**
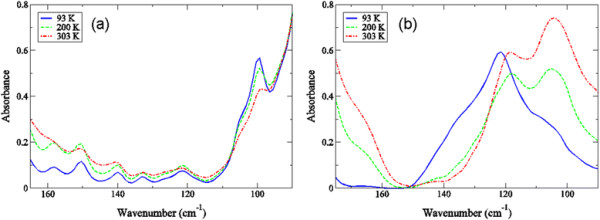
**Experimental THz spectrum of (a) the HHS and (b) the LHS of crambin in the 90 – 165 cm**^**−1**^**spectral region at 93 K (blue solid line), 200 K (green dashed line), and 303 K (red dot-dash-dotted line).**

Analysis of the origin of the modes detected in the experimental spectra in Figure 8a suggests that the underlying source of the prominent features, particularly in the LHS sample, may be attributed to protein specific interactions that promote long-rage communication within the protein 3-D structure. Principally, again relying on the MD simulation results, we find that correlated fluctuations involving amino acids within the central region of the LHS (Figure 8b) produce an efficient pathway through the interior of the molecule. Specifically, in Figure 8b we have plotted a graphical representation of the magnitude of the cross correlation coefficient (*C*_*ij*_*)* found in the LHS MD simulation using Equation (3) where the residues depicted have a *C*_*ij*_ value ≥ 0.35. Many of the residues participating in the network of fluctuations highlighted in Figure 8b include disulfide bonds as well as other hydrophilic amino acids. We do not observe this type of long-range communication in the HHS crambin 3-D structure. In Figure 8c – d, an examination of the nature of the fluctuations of specific residues involved in the long-range network of interactions detected in the LHS from the MD simulation indicate that the 105 cm^−1^ likely reflects a rotational component of the long-range interaction within the core of the molecule while the 120 cm^−1^ mode reflects a translational component. A subsequent experimental investigation of the modes in the same higher frequency spectral region (100 – 170 cm^−1^) as a function of temperature in Figure 9a – b supports this assessment. In the LHS experimental spectrum in Figure 9b, the previously identified mode at 105 cm^−1^ is nearly absent at 93 K, but continues to grow in amplitude as the transition temperature is reached and eventually crossed. The progression of the 105 cm^−1^ mode as a function of temperature indicates that it is characteristically anharmonic. The 120 cm-^1^ mode is only slightly altered in both intensity and frequency as the sample temperature is increased from 93 K up to 303 K suggesting that the band emanates from vibrational motion. Additionally, it is worth noting that in the LHS spectrum there is also a noticeable shoulder at approximately 140 cm^−1^ that is only apparent below the glass transition (93 K). The 140 cm^−1^ experimental peak is associated with a translational mode that is sustained by the α-helical hydrogen bonding network in the protein secondary structure and supported by interactions with the solvent [57],[58]. Returning to the point that the HHS spectrum has less prominent peaks in the equivalent spectral region (Figure 9a) is strongly suggestive of the fact that the probable associations that the protein has with the solvent molecules in the hydration shell are such that they do not promote large-scale protein fluctuations.

## Conclusion

In this work we have explored the THz time scale fluctuations in crambin both from below and above the glass transition temperature. In line with previous investigations [4],[13]-–[17],[29],[53],[59],[60], we have deduced that the solvent fluctuations on a picosecond time scale strongly influence the protein fluctuations on an equivalent time scale. Through association with the solvent, we find that a sample with only the first hydration filled (LHS) has large-amplitude, anharmonic fluctuations as the glass transition temperature is approached. The LHS interacts with solvent water with an open, chain-like network, and the solvent-protein interactions in LHS promote collective backbone modes that extend throughout the entire protein. The HHS interactions with the solvent do not support anharmonic fluctuations and the progression through the glass transition is somewhat unremarkable. Furthermore, the class of water structures in the HHS hydration shell that are available for the protein to interact appear to be quite dissimilar. And perhaps as a consequence, the types of surface interactions that HHS shares with the solvent include mostly localized side-chain torsions that do not change appreciably during the transition. In light of these findings, it is possible that the pattern that we have observed experimentally simply reflects the hydrophobic nature of crambin; but it may also serve as a model for understanding sub-nanosecond protein-solvent coupled interactions in globular proteins in a broader sense.

So far, we have proposed that a general explanation for the differences in the crambin spectra in the low frequency THz region is the manner in which the solvent couples to the protein low frequency modes. The standard model [29] of the glass transition describes a particle moving across a rugged energy landscape, and below the transition temperature the particle is confined within a potential well. Near and above the transition temperature, the particle is able to overcome some of the lower barriers, resulting in what is referred to as “β- relaxation processes”. The β-relaxation process in proteins involves cooperative backbone fluctuations on a picosecond time scale. It has been conjectured that the fast conformational fluctuations connected with the β-process are a precursor for overcoming larger barriers associated with a principal α-relaxation (large-scale protein conformational change) that occurs on a much longer time scale [61]. In the LHS spectra we find that the solvent coupling promotes large amplitude collective fluctuations in the protein when the transition temperature is reached. Indirect analysis of the water structure (Figure 6) in the LHS hydration shell has revealed an open water structure with a distinctive translational mode temperature dependency. It is through the dynamics of this less-ordered water structure that we surmise that the H-bonding network vibrations in the LHS support not only anharmonic fluctuations but also vibrational mode delocalization (vibrational modes that involve the entire protein) on a picosecond time scale. The HHS spectra reveal an entirely different story. The protein coupling with the solvent in HHS results in mostly localized, harmonic fluctuations in the ≤ 200 cm^−1^ spectral region. The basis for the localized fluctuations in HHS could be due to the overall rigidity of the H-bonding in the solvent network; or perhaps, a consequence of the protein being excluded from the aforementioned network and as an outcome unable to overcome barrier crossings. In actuality, the details pertaining to the interactions taking place within the extended solvent H-bonding network are beyond the scope of our current work on crambin and therefore, at this stage are mostly just speculation. The central issue from our analysis is that the nature of the protein-solvent coupling in HHS differs from LHS on the picosecond time scale. These distinctions appear to have significant ramifications for both the amplitude and characteristics of the low frequency protein modes detected in the THz experimental spectrum. The collective excitations that emerge after the glass transition in LHS may serve as a model for forming a better understanding about the types of motions that are generally triggered during the onset of the transition in hydrated globular proteins. Characterization of the THz time scale vibrational modes in the protein may also provide an opportunity to begin to elucidate the functional role of crambin.

## Methods

### Sample preparation

Crambin was purchased from GenScript (Piscataway, NJ). Salt and other contaminations were removed from the sample prior to the experiment, by acetone precipitation and re-suspended in a buffer consisting of 10 mM NaH_2_PO_4_ and 0.01 mM ethylenediaminetetraacetic acid at pH 7.0. The concentration of the protein sample was determined by UV absorbance using a standard curve derived from a series of dilutions at 280 nm. The crambin samples used in the experiments were initially prepared by diluting the stock protein sample to a concentration of 1 mg/ml. 20 μL (~20 μg) of the diluted sample was subsequently placed on a high resistivity silicon window and excess water was removed by applying a low, steady flow of N_2_ gas over the sample droplet for approximately 10 minutes. The resulting protein film was subsequently rehydrated by equilibrating the partially dried sample in a vacuum sealed container with the vapor pressure of a saturated salt solution at 20°C for a minimum of 5 days [62]. The low hydration crambin sample was hydrated with a saturated salt solution of NaCl while the fully hydrated crambin sample was hydrated with a saturated salt solution of K_2_SO_4_. The number of water molecules in the hydrated protein samples was estimated by thermal gravimetric analysis. Experimental measurements on hydrated, unoriented crambin film samples prepared from saturated salt solutions in this manner have revealed that samples equilibrated with a saturated salt solution of K_2_SO_4_ result in a hydration level of approximately 0.8 g/g. At this hydration level, the water molecules available in the hydration layer are sufficient for completing both the first and second hydration shell of the globular protein [63]. The sample prepared in the presence of a saturated solution of NaCl result in hydration level of approximately 0.25 g/g and in this case the number of water molecules available is adequate for hydrating both polar and non-polar residue surface groups in the protein [63].

The prepared film sample was placed in a sealed transmission cell consisting of two high resistivity silicon substrates and a saturated salt solution was placed at the bottom of the cell to ensure that hydration was maintained throughout the experiment. Experiments with D_2_O as the solvent were prepared in a similar manner except that the crambin sample was dissolved in D_2_O rather than water. The D_2_O solution sample was initially allowed to equilibrate for two days at 4°C and then the D_2_O film samples were prepared and allowed to equilibrate at room temperature for another 5 days prior to experiment.

### Molecular dynamics simulations

The molecular dynamics (MD) simulations were carried with the Gromacs package [64] version 4.5.3 using the all-atom OPS force field. A starting structure of the crambin configuration was initially downloaded from the protein databank (1EJG.pdb). For the low hydration sample the protein was initially hydrated with solvent molecules in a 3.8 Å shell surrounding the protein surface, which corresponds to 270 water molecules. The sample with higher hydration content consists of 854 water molecules and corresponds to a hydration shell that extends out to 8.0 Å from the protein surface. In the simulations, the SPC model of water was used. Energy minimization of the hydrated protein system was carried out by using a steepest descent method to a convergence tolerance of 0.001 kJ mol^−1^. The energy minimization was followed by a MD run with constraints for 200 ps in which an isotropic force constant of 100 kJ mol^−1^ nm^−1^ was used on the protein atoms. During the restrained dynamics simulation, the temperature and pressure of the system were kept constant by weak coupling to a modified velocity rescaled Berendsen temperature and pressure baths [65] and in all cases the protein and water molecules have been coupled to the temperature and pressure baths separately. The final output configuration from the MD simulation with constraints was used as the starting configuration for a 5 ns equilibration MD simulation run. Equilibration steps were performed with periodic boundary conditions. Configurations from the equilibration run were used as starting configurations for a series of 10 ns MD simulations. These final simulations were carried out with a 1 fs time step where the bonds between the hydrogen and the other heavier atoms were restrained to their equilibrium values with the linear constraints (LINCS) algorithm [66]. Particle mesh Ewald (PME) method [67] was used to calculate the electrostatic interactions in the simulation and was used with a real-space cutoff of 1.0 nm, a fourth order B-spline interpolation and a Fourier spacing of 0.12 nm. In the MD simulations with heavy water_,_ the water molecules were replaced with D_2_O.

The velocity autocorrelation function (VACF) of both atoms and molecules from the MD simulations were computed with the extended analysis tools that are included as part of the Gromacs software package [68]. The VACF is defined by

(1) $$C_{v}\left(\tau \right)=\frac{\langle v_{i}\left(\tau \right)\cdot v_{i}\left(0\right)\rangle }{\langle v_{i}{\left(0\right)}^{2}\rangle },$$

where *v* refers to velocity and *i* denotes an atom or molecule in the simulation system. Fourier transform of the VACF is used to project out the underlying frequencies of the molecular processes associated with the correlated motions detected in the simulation.

Dynamical fluctuations within the hydrogen bond network within the protein or between the protein and the solvent molecules have been characterized by use of a correlation function [69]

(2) $$C_{\mathrm{hb}}\left(\tau \right)=\frac{\langle hb_{i}\left(\tau \right)\cdot hb_{i}\left(0\right)\rangle }{\langle hb_{i}{\left(0\right)}^{2}\rangle },$$

which averages over hydrogen bond pairs, and has an either 0 or 1 (hb(τ) = [0,1]) for a particular hydrogen bond *i* at time *t*. In this analysis, a hydrogen bond is defined by using a geometrical criterion, where the center of mass distance is less than 3.5 Å, the r(O•••H) distance is smaller than 2.6 Å, and the ∠HO•••O angle is smaller than 30°. Other weak interactions (Van der Waals, electrostatic, etc.…) in the protein system are identified as contacts within an appropriate cut-off distance.

The extent to which amino acid fluctuations in the protein three-dimensional structure are correlated depends on the magnitude of the cross correlation coefficient (*C*_*ij*_) [70], which is given by

(3) $$C_{\mathrm{ij}}=\frac{\langle \Delta r_{i}\cdot \Delta r_{j}\rangle }{{\left({\langle \Delta r_{i}\rangle }^{2}{\langle \Delta r_{j}\rangle }^{2}\right)}^{1/2}},$$

where *i* and *j* represent two separate residues and ∆*r*_*i*_ and ∆*r*_*j*_ are the displacement vectors of *i* and *j* where the brackets represent ensemble averages. In the cross correlation of the residue fluctuations if *C*_*ij*_ *=* 1 then the fluctuations of *i* and *j* are completely correlated and if *C*_*ij*_ *= −*1 then the fluctuations of *i* and *j* are completely anti-correlated and if *C*_*ij*_ *=* 0 then the fluctuations of *i* and *j* are not correlated. In the graphical depiction of *C*_*ij*_ only the magnitude of correlated fluctuations between residues with a value greater than 0.25 are considered.

### THz spectroscopy experiments

The THz spectroscopy experiments were carried out on a Jasco FTIR—6000 series spectrometer. The crambin film samples were collected with a liquid helium cooled bolometer in the 15–250 cm^−1^ spectral range. The sample cell used in the experiments contained a 0.006 mm thickness polytetrafluoroethylene spacer (Specac Ltd., U.K.) and for each transmission measurement a 25 mm diameter region of the protein film sample was illuminated with the THz beam to determine the absorbance. To calculate the absorption spectrum, a background spectrum was initially collected (*I*_0_). A subsequent measurement of the prepared sample in single beam configuration was also collected. To normalize the infrared measurement, the single beam of the sample (*I*) is divided by the background signal (*I*_0_). The result is a percent transmittance spectrum $\left(\%T=\frac{I}{I}\times 100\right)$. The absorbance spectrum was calculated from the transmittance spectrum by using the relationship

(4) $$A=-\log\left(\frac{\%T}{100}\right),$$

To maintain the hydration level of the protein film during the experiment, the sample was placed in a sealed transmission cell consisting of two silicon windows. Reversibility of the temperature response of the protein sample, in terms of absorption features and intensity, was one criterion used to verify that the seal was maintained throughout the experiment. In the spectral measurements presented each scan consists of 16 averaged scans and the infrared data was collected with a spectral resolution of 4 cm^−1^ with an error of less than 2.0% between the individual scans used for averaging with the greatest error being found near the edges of the detection limits of the beam splitter. The 15–100 cm^−1^ THz spectra were collected with a 25 micron beam splitter while the data in the 100–250 cm^−1^ spectral region was collected with a 12 micron beam splitter. The temperature of the samples was varied using a SPECAC variable temperature cell. Using a combination of refrigerant and the control from the built-in temperature cell-block heaters, the temperature of the sample could be adjusted from −190°C to 30°C with stability of ± 0.1°C from the set temperature.

## Competing interests

The author declares that she has no competing interests.

## Authors’ contributions

KW devised, implemented, and performed all of the experiments and the simulations presented and additionally analyzed the data. KW also wrote the paper.
